# Propagation of Cannabis for Clinical Research: An Approach Towards a Modern Herbal Medicinal Products Development

**DOI:** 10.3389/fpls.2020.00958

**Published:** 2020-06-26

**Authors:** Suman Chandra, Hemant Lata, Mahmoud A. ElSohly

**Affiliations:** ^1^ National Center for Natural Products Research, School of Pharmacy, University of Mississippi, Oxford, MS, United States; ^2^ Department of Pharmaceutics and Drug Delivery, School of Pharmacy, University of Mississippi, Oxford, MS, United States

**Keywords:** cannabis, micropropagation, vegetative propagation, tetrahydrocannabinol, cannabidiol

## Abstract

Cannabis has been reported to contain over 560 different compounds, out of which 120 are cannabinoids. Among the cannabinoids, Δ^9^-tetrahydrocannabinol and cannabidiol are the two major compounds with very different pharmacological profile and a tremendous therapeutic potential. However, there are many challenges in bringing cannabis from grow-farms to pharmaceuticals. Among many, one important challenge is to maintain the supply chain of biomass, which is consistent in its cannabinoids profile. To maintain this process, male plants are removed from growing fields as they appear. Even with that practice, still there are fair chances of cross fertilization. Therefore, controlled indoor cultivation for screening, selection of high yielding female plants based on their cannabinoids profile, and their conservation and multiplication using vegetative propagation and/or micropropagation is a suitable path to ensure consistency in biomass material. In this chapter, the botany and propagation of elite cannabis varieties will be discussed.

## Introduction

For thousands of years, cannabis is being cultivated to be used in day today need such as food, medicine, oil, textile fiber etc. The origin of this plant can be tracked back in China, wherefrom the plant made its way to the rest of the world.

**Figure 1:**
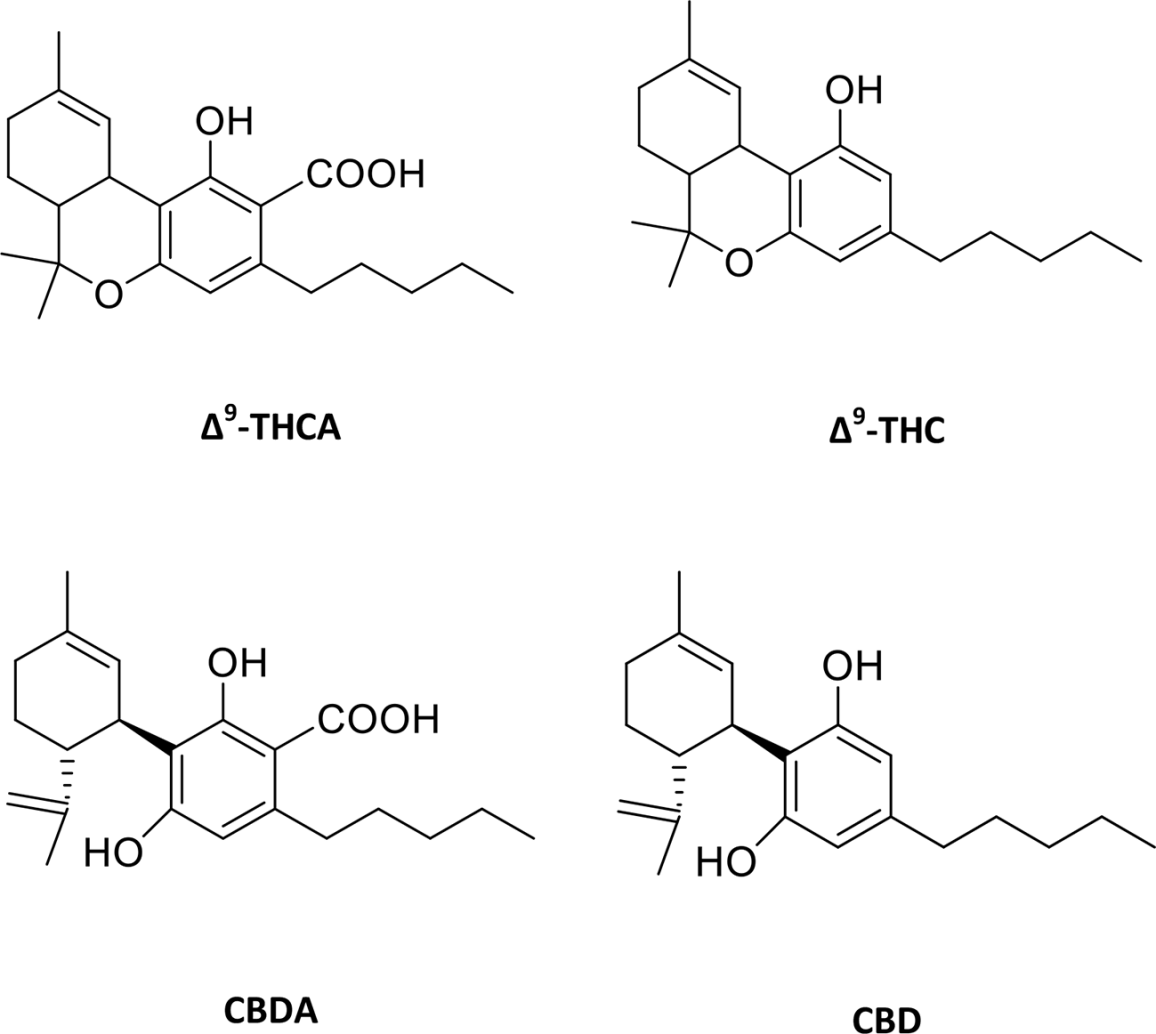
Molecular structures of major phytocannabinoids. Δ^9^-tetrahydrocannabinolic acid (Δ^9^-THCA), Δ^9^-tetrahydrocannabinol (Δ^9^-THC), cannabidiolic acid (CBDA) and cannabidiol (CBD).

Traditionally, the plant cannabis has been used to treat a wide variety of ailments such as asthma, epilepsy, fatigue, glaucoma, pain, and rheumatism (Mechoulam et al., 1976; Zuardi, 2006). Cannabis derivatives have also been reported to help in HIV/AIDS and multiple sclerosis (Pryce and Baker, 2005; Abrams et al., 2007). *Cannabis sativa* is the natural source of cannabinoids and Δ^9^-tetrahydrocannabinol (Δ^9^-THC) is the primary psychoactive agent. This compound is produced as an acid (Δ^9^-tetrahydrocannabinolic acid, Δ^9^-THCA, **Figure 1**) in plant and undergoes decarboxylation with age or heating to form Δ^9^-THC. The other interesting compound in cannabis is cannabidiol (CBD, **Figure 1**), which is a non-psychoactive compound and reported to be useful in the treatment of seizures and epilepsy, specifically for the intractable pediatric epilepsy (Mechoulam and Carlini, 1978; Cunha et al., 1980).

Cannabis is also a big source of natural fiber. Earliest cultivation of hemp can be tracked back to the Neolothic Age in China, where it was mainly grown for ropes, paper, and textiles fiber. Nowadays, cannabis is used in making varieties of products such as composites, health foods, cosmetics, clothing, biofuels, and more (Small, 2015).

## Complex Chemistry

The first compound that was isolated from cannabis was cannabinol (CBN, Wood et al., 1899). Its structure was determined much later in 1930s and 40s (Cahn, 1932; Adams et al., 1940a). CBD was isolated in 1940 and its *molecular structure was elucidated in 1963* (Adams et al., 1940b; Mechoulam and Shvo, 1963). Whereas, isolation of Δ^9^-THC was reported in 1964 (Gaoni and Mechoulam, 1964). The number of compounds isolated from cannabis has been continually increasing. Most recent review shows the plant to be rich in secondary metabolites, with more than 560 constituents reported (ElSohly and Slade, 2005; ElSohly and Gul, 2014; Radwan et al., 2017). Out of which, 120 are cannabinoids those are distributed among more than ten subclasses namely, Δ^9^-THC, Δ^8^-THC, CBD, CBG, CBN, CBND, CBE, CBL, CBT, and miscellaneous types.

**Figure 2:**
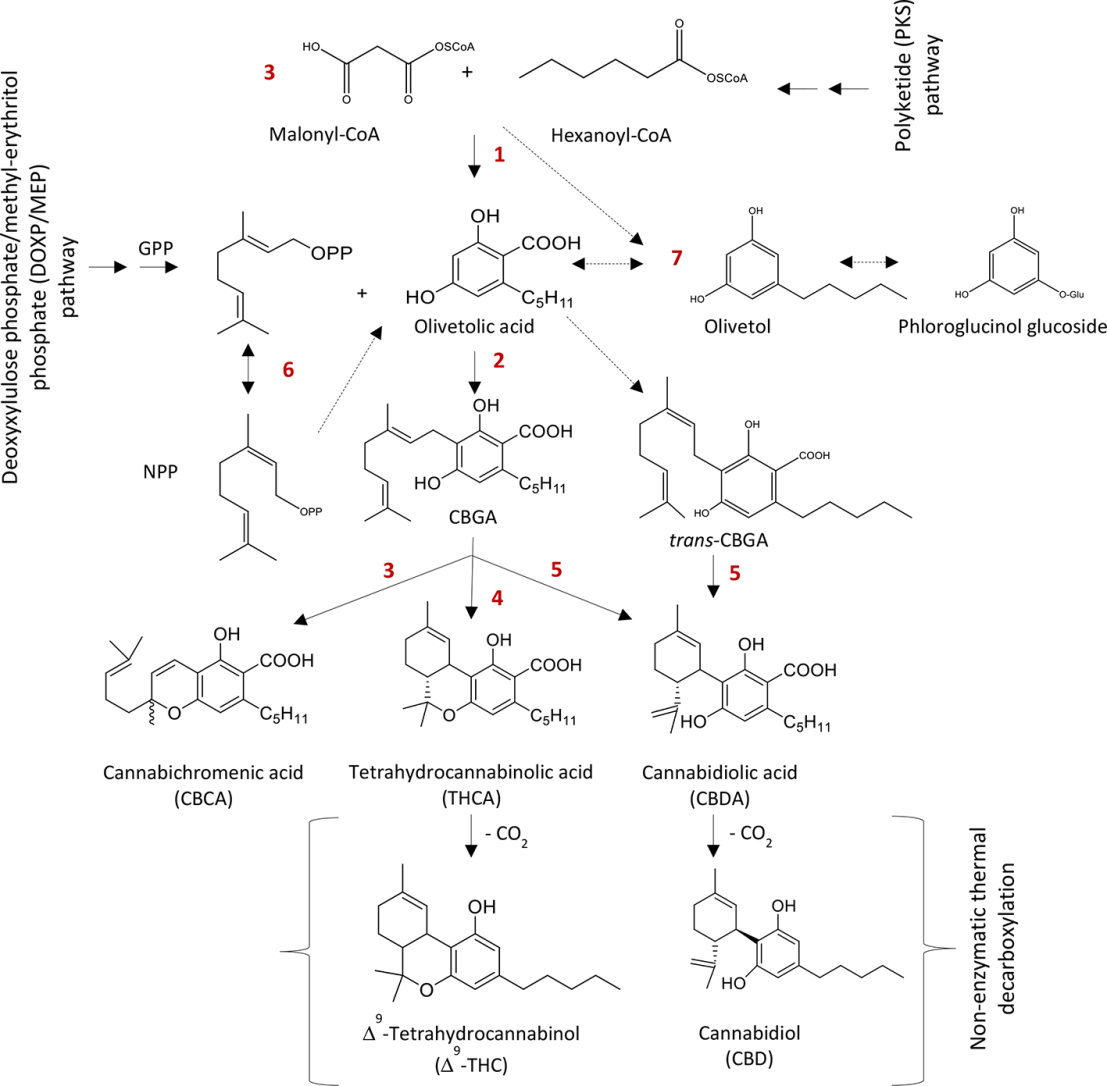
Biosynthesis of major phytocannabinoids. Δ^9^-THC and CBD. 1: Polyketide synthase (PKS), 2: Cannabigerolic acid (CBGA) synthase, 3: Cannabichromenic acid (CBCA) synthase, 4: Δ^9^-Tetrahydrocannabinolic acid (Δ^9^-THCA) synthase, 5: Cannabidiolic acid (CBDA) synthase, 6: Isomerase and 7: Olivetol synthase. GPP: Geranyl diphosphate and NPP: Neryl diphosphate.

A schematic diagram of the cannabinoids biosynthesis is shown in **Figure 2**. In the cannabis plant, cannabinoids are normally present in the acid forms such as THCA and CBDA (Shoyama et al., 1975; Fellermeier and Zenk, 1998) and turn in to neutral form after exposure to heat. The cannabinoids and their precursors are synthesized from two different pathways, the polyketide pathway (PKS) and the deoxyxylulose phosphate/methyl-erythritol phosphate (DOXP/MEP) pathway (Shoyama et al., 1975; Fellermeier et al., 2001). Geranyl diphosphate (GPP) and olivetolic acid (OLA) are synthesized from the DOXP/MEP and PKS pathways, respectively. GPP and OLA in combination form cannabigerolic acid (CBGA) through geranyl diphosphate:olivetolate geranyltransferase (GOT, Fellermeier and Zenk, 1998). Cannabigerolic acid is common substrate for CBDA synthase (Taura et al., 2006), Δ^9^-THCA synthase (Taura et al., 1995) and CBCA synthase (Morimoto et al., 1998), which ultimately form cannabidiolic acid CBDA, Δ^9^-THCA and CBCA, respectively (Morimoto et al., 1999; Sirikantaramas et al., 2007).

## Classification Debate

**Figure 3:**
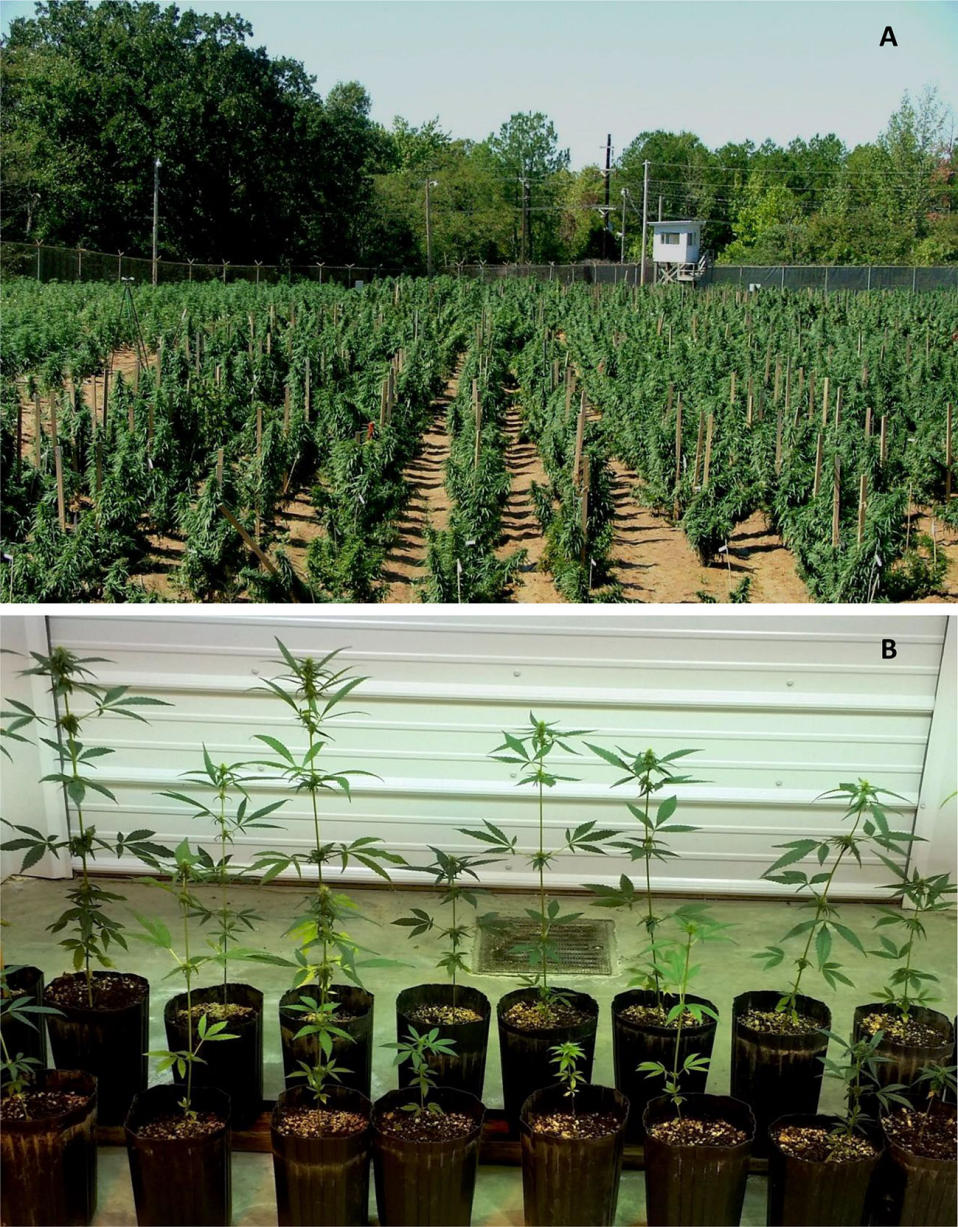
Representative cannabis varieties, **(A)** Drug type variety and **(B)** Fiber type variety.

Based on the plant morphology, cannabis can be characterized in two distinct groups, drug type and fiber type. Fiber type varieties grow skinny and tall with very few branches whereas, drug type varieties grow bushy, form a Christmas tree like shape with big branches at the lower part of the stem (**Figure 3**).

Cannabis can be classified in different varieties/groups qualitatively and quantitatively based on the chemical profile content (Mondolino et al., 2003). The ratio of THC and CBD in the leaves and the flowers of the plant is generally used as a marker to classify cannabis varieties. According to Fetterman et al. (1971), varieties having high THC and low CBD (THC/CBD > 1) were characterized as drug type otherwise (THC/CBD < 1) fiber type variety. Whereas, Small and Beckstead (1973a; 1973b) distinguished *C. sativa* in three phenotypes with an additional class containing THC~CBD. Further, a separate class of cannabis phenotype with high CBG was characterized by Fournier et al. (1987).

Considering the botanical variations, taxonomists have described cannabis variously. A number of reports proposed cannabis as a polytypic [multiple-species, Hillig (2004; 2005), McPartland and Guy (2004) and Clarke and Merlin (2013)] whereas others suggest as a single genus, (monotypic) but highly polymorphic species, *Cannabis sativa* L. (Small, 1975a; Small, 1975b; Small and Cronquist, 1976; Small, 2015). Currently, cannabis is considered to belong to one genus and one single, highly diverse species, *Cannabis sativa* L.

## Botanical Drug Development Approaches

Plants have been used as a medicine in all cultures since millennia. To develop natural products as a single molecule drug in modern medicine is costly and also, time consuming. Therefore, learning from traditional healthcare systems such as Ayurveda and Traditional Chinese Medicine (TCM), scientific focus is being directed to the development of botanical drugs (total plant extracts) used for the treatment of specific disease conditions. In this regard, US food and drug administration (FDA) has developed strict guidelines in 2006 for the development of “Botanical Drugs” products.

In the case of cannabis, variability in the botanical aspects translates into variability in the chemical makeup and the ratios of the different constituents for the different varieties. It follows that the pharmacological activities of the different varieties of cannabis must be different. Therefore, when one speaks about medicinal cannabis or medicinal cannabis preparations, it has to be chemically defined with specific therapeutic activity. In terms of the product development, any preparation of cannabis (plant material or extract) would be considered a botanical drug and must be fully characterized. Not only that the chemical makeup of the product would have to be defined, but one would have to show consistency in the chemical composition from one batch to the other. Furthermore, botanical drugs have to be approved for specific medical conditions through clinical trials. The most notable example of botanical drug from cannabis is Sativex^®^, also marketed with the name of nabiximols, developed by G.W. Pharma, which is a combination of THC and CBD extracts in equal ratio (1:1).

Another example is cannabis preparations sold by “Bedrocan”, a Netherlands based cannabis company. In Netherlands, the company sells chemically characterized cannabis biomass (buds) to patients through pharmacies with a valid doctor’s prescription. Whereas, these products are directly available in Canada (without prescription). Bedrocan has three high THC variety-based products namely Bedrocan^®^, Bedica ^®^ and Bedrobinol^®^, one (Bediol^®^) from intermediate variety (THC~CBD) and one (Bedrolite^®^) from high CBD variety. All these products are well characterized based on specific THC, CBD, and terpenes content. Although the company is certified in Europe, their products are not tested for specific disease conditions and not approved or accepted in the USA.

On the other hand, there are several formulations/drugs/cannabis preparations/products on the market (available in different States of America and on the internet) claiming their use for curing several disease conditions without any scientific proof of clinically efficacy. Not following the requirements of a true botanical drug means that the patients basically do not know what they are getting, with the possibility of dangerous side effects and possible exacerbation of their medical condition.

Cannabis botanical formulations are considered by many as more effective than the individual cannabinoids citing the “entourage” effect as the reason (Ben-Shabat et al., 1998; Mechoulam and Hanus, 2000). That is, the contribution of other cannabis constituents, such as other cannabinoids, terpenes and flavonoids, provide synergetic effects with the major cannabinoid’s activity. However, this assertion has been clinically proven.

## Regulatory Aspects of Cannabis Cultivation

The process of Plant based drug development face unusual challenges at every step from cultivation, harvesting, and processing to quality and consistency of biomass product. Cannabis in particular, faces a significant additional complexity due to being characterized as a schedule I drug.

In the United States, individual states have regulated cannabis through state legislation. Many states have legalized cannabis only for medicinal purposes but some of them have opened it for both medicinal and recreational uses. As this article is being written, 33 US states and DC have legalized cannabis for medical purposes and among them, 10 states and DC have opened it for both medical and recreational purposes. While cultivation of cannabis in these states is perfectly legal under the state laws, it is still illegal under the federal regulation. This creates an unusual situation for an authentic drug development. If a pure/botanical drug is developed under the federal regulation, it goes through a strict review by FDA. That covers all safety and efficacy issues of that product. However, medications/remedies under states legislations are bypassing all the FDA safety barriers.

## Cannabis: A Dioceous Plant

Cannabis is normally a dioecious plant. At the early (juvenile) stages of plant life cycle it is difficult, in fact impossible to discriminate morphologically between male and female plants. Some molecular techniques are reported to differentiate between male and female’s plant at early growth stage (Sakamoto et al., 1995; Flachowsky et al., 2001; Törjék et al., 2002; Sakamoto et al., 2005; Techen et al., 2010). These techniques however, have limited practical applications in case of a large-scale cultivation.

Cannabis is a wind pollinated species. If grown from seed, roughly 50% of the plants will be males and 50% females. To maintain consistency in cannabinoids profile and content in the final product (biomass or resin), cannabis cultivation is currently mostly carried out through vegetative propagation. The quality and quantity of biomass produced is highly variable due to the allogamous nature of the cannabis plant. To maximize cannabinoids production and to maintain consistency in cannabis biomass production female plants are preferred over male plants. Male plants release pollen grains that set seeds in female plants which affects cannabinoids production negatively. Further, if several varieties of cannabis are grown together through seeds, the final biomass product of those plants will not be consistent due to cross pollination. Therefore, male plants are removed from cultivation area as soon they appear to avoid cross fertilization. In modern cultivation practices, seed free (sinsemilla) cannabis plants are preferred for maximizing the production of phytocannabinoids.

## Screening and Selection of Elite Clones for Mass Propagation

As stated above, Cannabis is chemically complex and a highly variable plant due to its cross-fertilization nature. Different varieties of cannabis plants contain a wide-range of cannabinoids and other chemical components ranging from hemp (low in THC, <0.3%) to highly potent drug type varieties with THC far exceeding 10% in some varieties. These levels are mostly determined by the plant genetics and influenced by several parameters such as growth environment, fertilization, harvesting time etc. (Valle et al., 1978; Hemphill et al., 1980; de Meijer et al., 1992; Pate, 1994; BóCsa et al., 1997; de Meijer et al., 2003; Chandra et al., 2008; Chandra et al., 2010; Mendoza et al., 2009). Variations in cannabinoids content among different plant parts have also been reported by Hemphill et al. (1980). For a pharmaceutical drug development, a stable source of biomass which is consistent in the production of secondary metabolite and a standardized growing protocol is of utmost importance. In case of cannabis, a batch to batch consistency in cannabinoids profile and content in particular, is very important for the development of a pharmaceutical or botanical drug. This can be achieved by selecting and germinating a desirable seed lot, removing male plants from growing area as they appear (since male flowers are morphologically different and appear earlier than female flowers, they are easy to recognize), making backup cuttings from female plants (kept in vegetative environment, 18 h photoperiod) and letting female plants flower (12 h photoperiod) up to maturity. Biomass sample from fully mature plants were then taken and tested for their cannabinoids profile and content. Based on cannabinoids analysis high yielding female mother plants are identified and their backup cuttings are used for the future cultivation. Monitoring cannabinoids content for genetic material selection could be carried out by one of several analytical methods such as GC-FID (Ross and ElSohly, 1995), HPLC (Gul et al., 2015), UPLC (Wang et al., 2018).

**Figure 4:**
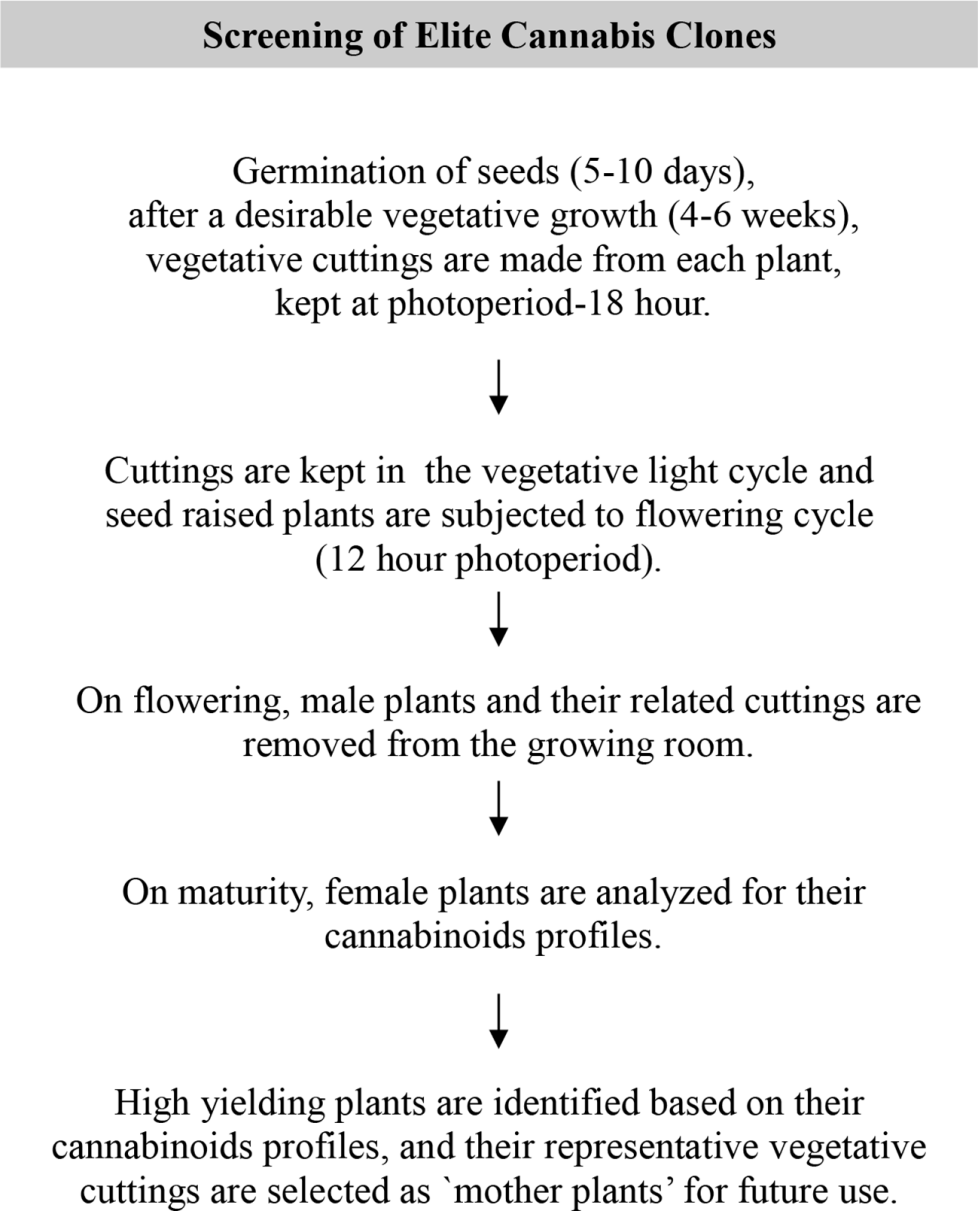
Schematic diagram of screening of elite cannabis clones.

Once high potency mother plants are identified and selected based on their cannabinoids profile they are multiplied asexually, yielding identical clones using conventional (vegetative cutting) and/or biotechnological tools (tissue culture) to ensure a batch to batch consistency in the final product. A schematic diagram of screening and selection process of elite mother plants is shown in **Figure 4**.

## Cannabis Horticulture

### Plant Life Cycle in Nature

Cannabis is an annual plant. In nature, sprouting of seeds starts during early spring (March–April). Plants continue to grow vegetatively during long days. It starts flowering as days start becoming shorter and set seeds before the arrival of winter. Some auto-flowering varieties flower on their own rhythm, not depending on the photoperiod. During the flowering stage, big leaves start yellowing and start falling from plants. On maturity, flowers/inflorescence are eventually developed in the form of buds. The maturity of plants depends upon the variety and the geographical area. Some early maturing varieties are ready to harvest by August-September and others get ready during October-November. The male plants if not removed at early stage, normally die after setting their pollens. Buds are harvested for phytocannabinoids and seeds for future crop or for seed oil. Plants eventually die if not harvested. Cannabis crop can be easily grown indoor or outdoor.

### Indoor Cultivation

**Figure 5:**
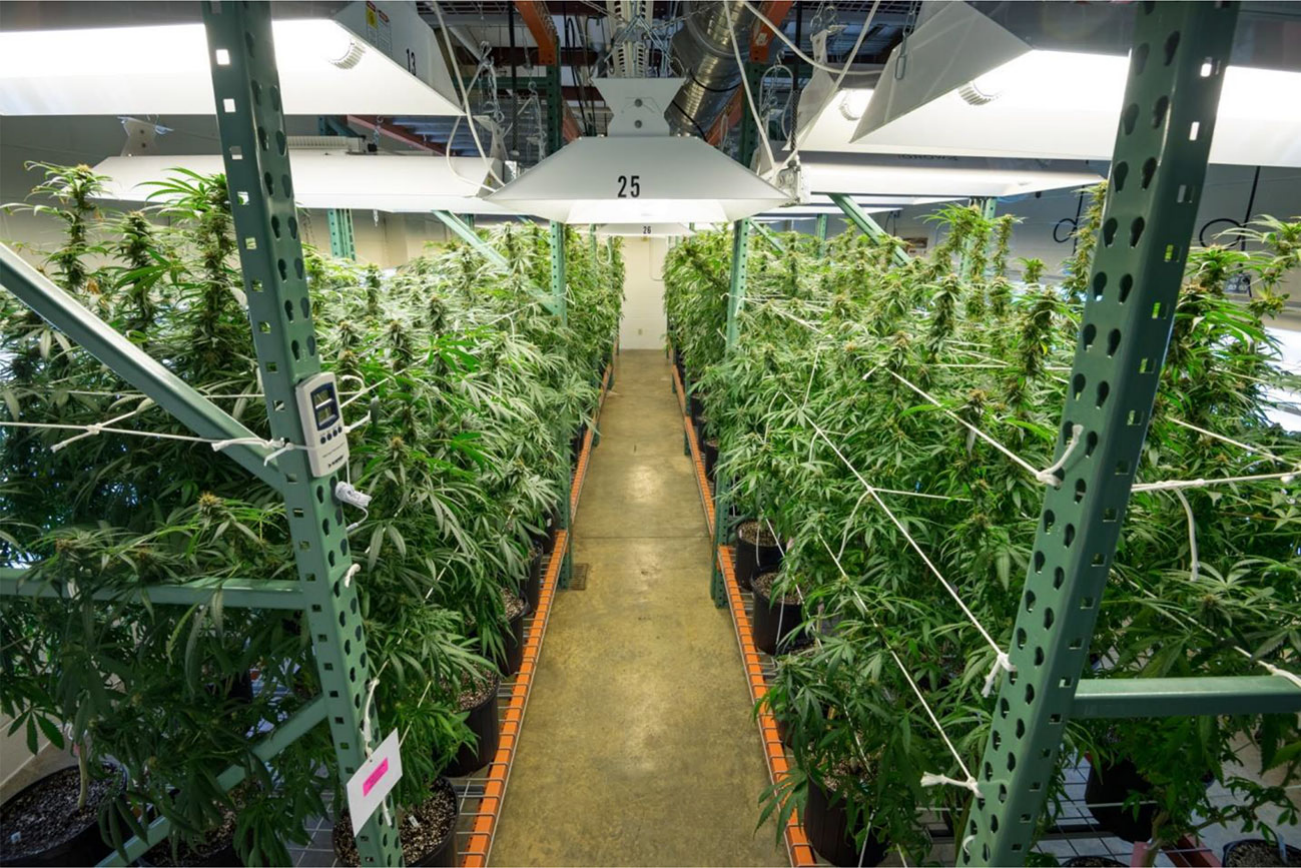
Indoor cultivation of *Cannabis sativa* L.

Depending upon the choice, three to four cycles of crop can be produced indoor annually. Light (quality and quantity), photoperiod, temperature, relative humidity, air circulation, and carbon dioxide level are the major environmental parameters that play an important role in cannabis cultivation. Under indoor climatic controlled conditions screened and selected high yielding female clones can be mass-propagated in soil or in liquid medium (Chandra et al., 2008; Chandra et al., 2015, **Figure 5**).

#### Vegetative Propagation

For vegetative propagation (in soil or in soilless medium), a sturdy, fresh, and healthy stem cutting containing one or more nodal segments and leaves, is used. To maximize the surface area of the rooting space, a diagonal cut is made on stem below a node. Cuttings are then immediately placed in clean water to prevent formation of air bubbles in the stems. Rooting hormone (such as “Garden Safe”, www.gardensafe.com, that contains 0.1% Indole-3-butyric acid, IBA) is applied to the base of cutting to promote rooting before planting in soil. Similarly, in hydroponics system 8–10-inch tall cuttings with one or more nodes are dipped in rooting hormone and wrapped by rock-wool or planted in hydrotone clay ball that serves as supporting medium. In both systems (soil or hydroponics) rooting initiates in 2–3 weeks. Eight-week old rooted plants are normally ready to be transplanted in bigger regular size pots.

To maintain vegetative growth plants are exposed to long photoperiods (normally > 12 h, preferably 18 or in some cases 24 h, Chandra et al., 2008; Chandra et al., 2015; Potter, 2015). Plants are supplied with vegetative fertilizer formula, comparably with higher nitrogen than flowering stage. Plants are exposed to a photoperiod <12 h to induce flowering. Once exposed to the flowering light cycle, plants start flowering within 10–15 days and ultimately form buds with highest cannabinoids content in overall plant life cycle (Chandra et al., 2008; Chandra et al., 2015). Depending on the variety, plants normally mature in 6 to 9 weeks. Length of vegetative growth period can be increased or decreased based on the plant growth and biomass yield/plant projected.

To achieve optimum growth and productivity, cannabis is best grown under (depending on genetics) 25 to 30°C growth temperature, high light intensity, and higher CO_2_ concentration (Chandra et al., 2008; Chandra et al., 2011a). Our studies show that cannabis exhibits higher rate of photosynthesis at high photosynthetic photon flux density (PPFD, ~1500 µmolm^2^s^-1^), which is typically sunny summer day in Mississippi (Chandra et al., 2008; Chandra et al., 2015). Further, about a 50% increase in the rate of photosynthesis was observed under doubling of CO_2_ concentration as compared to ambient CO_2_ concentration (Chandra et al., 2008; Chandra et al., 2011b). Higher humidity is generally ~60–75% is recommended at the young vegetative stage of plants whereas a lower range of 50 to 55% is recommended during the flowering stage.

#### Micropropagation

**Figure 6:**
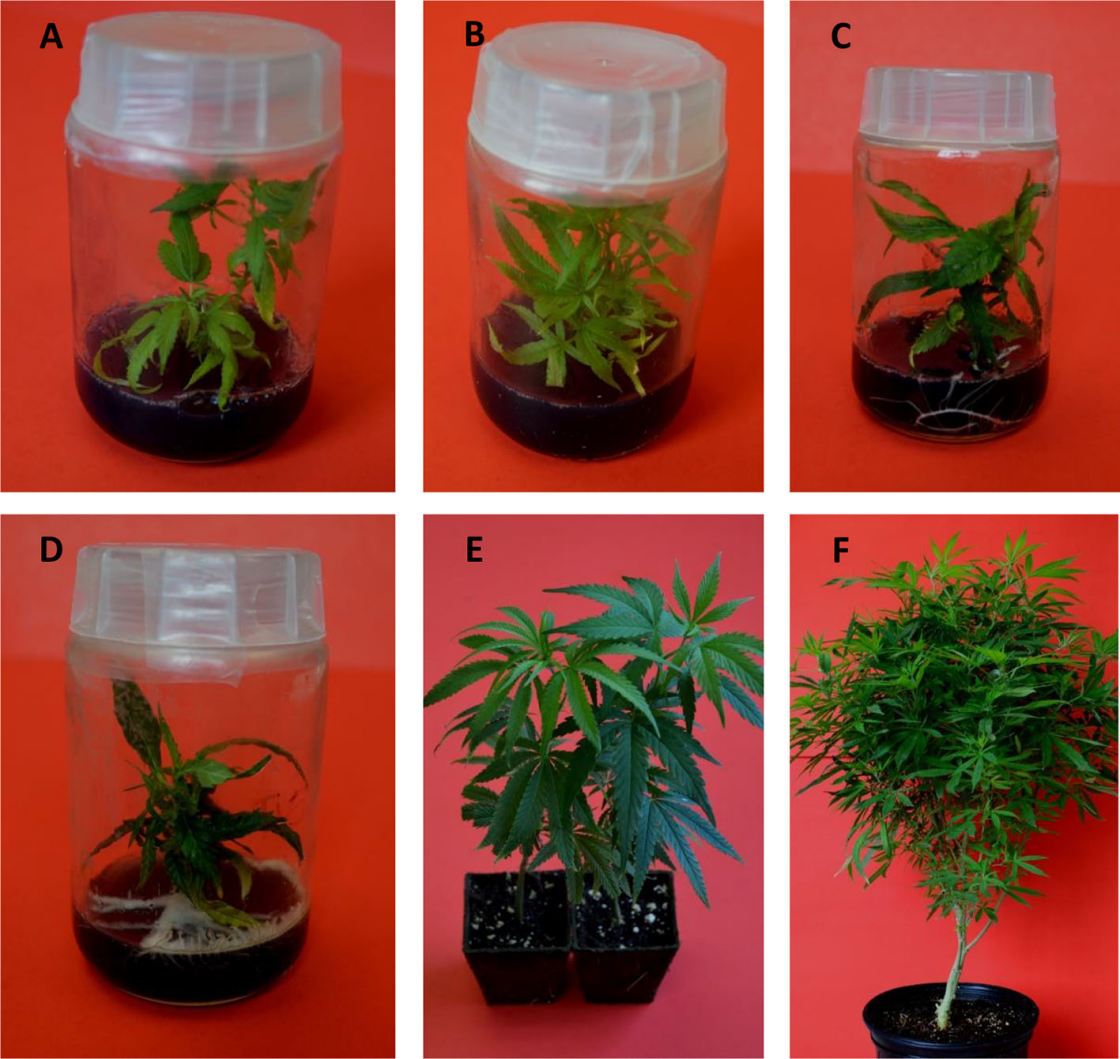
Micropropagation of *Cannabis sativa* L. **(A, B)** Formation of shoots, **(C, D)** Initiation of rooting, **(E)** Well acclimatized rooted plants in jiffy pots, and **(F)** Fully grown *in vitro* raised plants at vegetative stage.

Micropropagation has been used for decades for propagating plants of medicinal and agricultural value. A large number of medicinal plants required by the pharmaceutical industry are micropropagated on commercial scale include *Atropa belladonna, Cassia angustifolia, Catharanthus roseus, Cephaelis ipecacuanha, Datura innoxia, Digitalis purpurea, Eucalyptus globulus, Ocimum sanctum, Papaver somniferum, and Plantago ovata*, to name a few (Chaturvedi et al., 2007). Limited work on micropropagation of *Cannabis sativa* has been done prior to last decade. In our laboratory at The University of Mississippi, efficient protocols for production of clonal plants of *C. sativa* have been developed using nodal segments as well as leaf discs (**Figure 6**, Lata et al., 2009a; Lata et al., 2009b; Chandra et al., 2010; Lata et al., 2010; Lata et al., 2016). The protocols developed would be helpful for large scale mass propagation of elite cannabis varieties and will allow the breeders saving time and resource in mass propagation of healthy and uniform cannabis plants.

### Outdoor Cultivation

**Figure 7:**
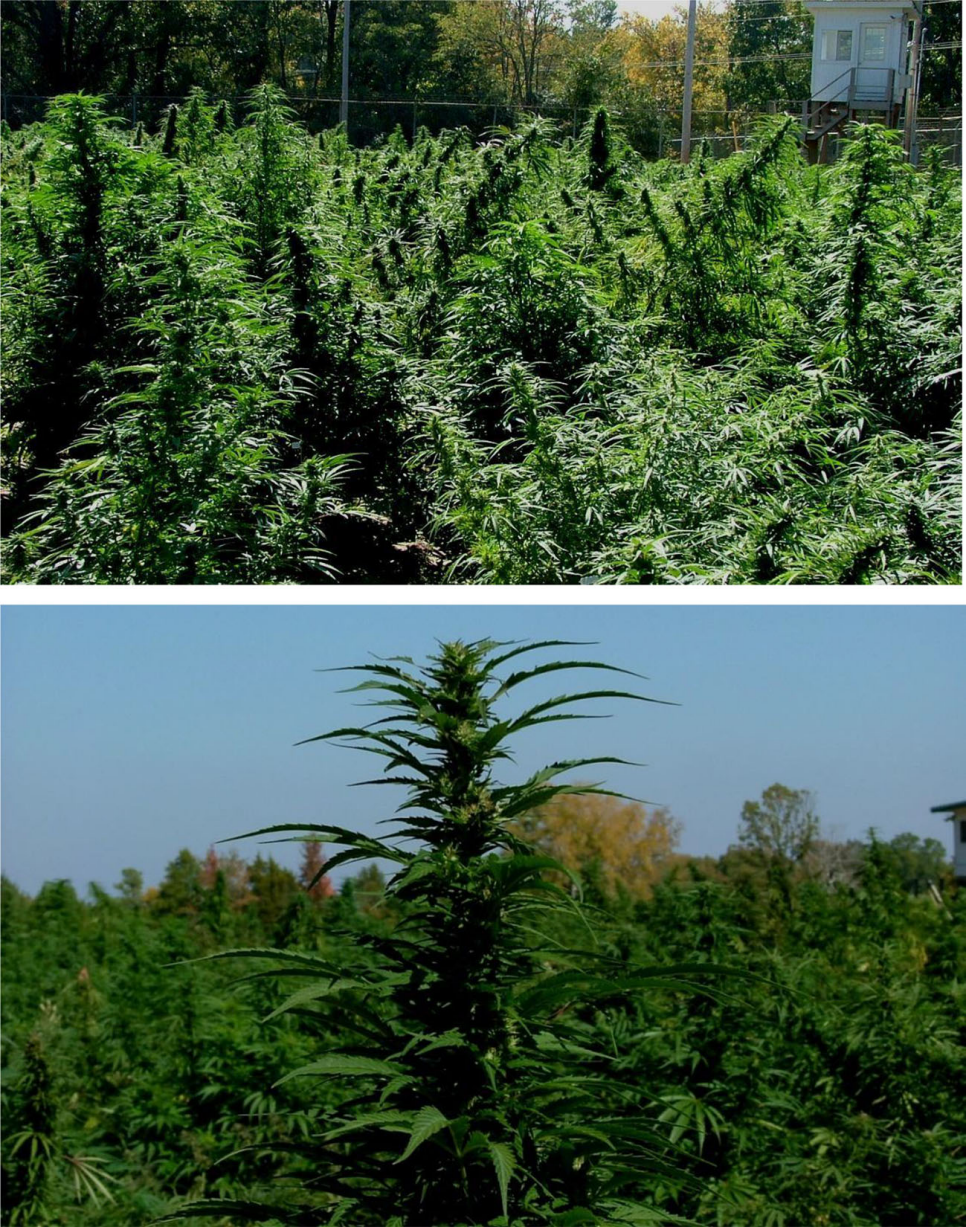
Outdoor cultivation of *Cannabis sativa* L.

Cannabis is an annual herb. It grows vegetatively during summertime due to long days and flowers during fall/winter with days turning shorter (**Figure 7**). If not harvested, plants go to senescence and eventually die. Cannabis can be grown by planting seeds directly in the ground, by planting them in biodegradable jiffy pots for germination and then planting the seedlings in the ground or by planting rooted cuttings. A big disadvantage of growing from seeds is that half of the crop will be male plants. To avoid pollination and seed production, male plants are removed from the field which makes almost half of the field empty. To avoid this situation, rooted cuttings of screened and selected high yielding female plants are preferred for the production of biomass due for consistency of the cannabinoids profile.

Determination of plant maturity and optimum harvesting time is a crucial step of any crop. With cannabis, optimum harvesting time can be determined by visual observation and/or cannabinoids content analysis. Cannabinoids content increase with plant growth. With the onset of flowering, a tremendous increase in cannabinoids content is observed as compared to the vegetative stage. The plants are harvested at peak flowering stage, following one of two methods. In one of the methods, whole plants are harvested and processed, and in the second method, selected mature buds are harvested first and more time is given to lower branches to form buds to maximize the harvest.

Once harvested, branches are separated from the main stem and cut into small pieces before drying. Dried or dead leaves are removed before drying. Depending upon the harvest size, drying of biomass can be done either by hanging the whole plants or large branches upside down in a well ventilated barn until drying or using an industrial grade “forced-hot air dryers” (similar to tobacco processing) used for large scale drying.

Adequately dried biomass is stored at 18–20 °C for short term and at ≤-10 °C for long term storage in the dark to avoid oxidation. In a study, Trofin et al. (2012) have shown a steady decay of Δ^9^-THC content in cannabis biomass for up to four years stored at room temperature (~22^0^C). The decay in THC was reported more pronounced under light conditions as compared to that stored in the dark. Cannabis biomass for scientific investigations is used either as the processed plant material or is used as the starting material for the preparation of extracts.

## Data Availability Statement

The datasets generated for this study are available on request to the corresponding author.

## Author Contributions

SC and HL wrote and ME reviewed the manuscript.

## Conflict of Interest

The authors declare that the research was conducted in the absence of any commercial or financial relationships that could be construed as a potential conflict of interest.

## Abbreviations

Δ^8^-THC, Δ^8^-Tetrahydrocannabinol; Δ^8^-THCA, Δ^8^-Tetrahydrocannabinolic Acid; Δ^9^-THC, Δ^9^-Tetrahydrocannabinol; Δ^9^-THCA, Δ^9^-Tetrahydrocannabinolic Acid; CBC, Cannabichromene; CBCA, Cannabichromenic Acid; CBD, Cannabidiol; CBDA, Cannabidiolic Acid ; CBE, Cannabielsoin; CBEA, Cannabielsoinic acid; CBG, Cannabigerol; CBGA, Cannabigerolic acid; CBL, Cannabicyclol; CBLA, Cannabicyclolic acid; CBN, Cannabinol; CBNA, Cannabinolic acid; CBND, Cannabinodiol; CBT, Cannabitriol; DOXP, Deoxyxylulose phosphate; GOT, Geranyl diphosphate:olivetolate geranyltransferase; GPP, Geranyl diphosphate; NPP, Neryl diphosphate; OLA, Olivetolic acid; MEP, Methyl-erythritol phosphate; OLA, Olivetolic acid; PKS, Polyketide synthase.
